# Pleural Aerobic Culture Status, Clinical Phenotypes, and In-Hospital Outcomes in Adult Pleural Infection: A Retro-Spective Cohort Study

**DOI:** 10.3390/medicina62102013

**Published:** 2026-10-18

**Authors:** Madalina Alexandra Balica, Catalin Balan, Norbert Wellmann, Lavinia Balan, Norberth-Istvan Varga, Daniela Teodora Marti, Razvan Susan, Ovidiu Rosca, Lavinia Davidescu, Ovidiu Fira-Mladinescu

**Affiliations:** 1 Doctoral School, “Victor Babes” University of Medicine and Pharmacy, Eftimie Murgu Square 2, 300041 Timisoara, Romania; madalina.balica@umft.ro (M.A.B.); bcatalin43@yahoo.com (C.B.); 2 Pulmonology Clinic, Clinical Hospital of Infectious Diseases and Pneumophthisiology Dr. Victor Babeș, 300310 Timisoara, Romania; norbert.wellmann@umft.ro; 3 Center of Research and Innovation in Personalized Medicine of Respiratory Diseases (CRIPMRD), “Victor Babes” University of Medicine and Pharmacy, 2 Eftimie Murgu Square, 300041 Timisoara, Romania; mladinescu@umft.ro; 4 Department of Obstetrics and Gynecology, “Victor Babes” University of Medicine and Pharmacy, Eftimie Murgu Square, No. 2, 300041 Timisoara, Romania; lavinia.balan@umft.ro; 5 Department of Nursing, “Victor Babes” University of Medicine and Pharmacy, Eftimie Murgu Square, No. 2, 300041 Timisoara, Romania; norberth.varga@umft.ro; 6 Multidisciplinary Research Center on Antimicrobial Resistance (MULTI-REZ), Microbiology Department, “Victor Babes” University of Medicine and Pharmacy, Eftimie Murgu Square No. 2, 300041 Timisoara, Romania; 7 Department of Biology and Life Sciences, Faculty of Medicine, Vasile Goldis Western University of Arad, 310025 Arad, Romania; marti.teodora@uvvg.ro; 8 Department of Family Medicine, Centre for Preventive Medicine, “Victor Babes” University of Medicine and Pharmacy, Eftimie Murgu Square No. 2, 300041 Timisoara, Romania; razvansusan@umft.ro; 9 Department XIII, Discipline of Infectious Diseases, “Victor Babeș” University of Medicine and Pharmacy, Eftimie Murgu Square 2, 300041 Timisoara, Romania; ovidiu.rosca@umft.ro; 10 Medical Department, Faculty of Medicine and Pharmacy, University of Oradea, 410073 Oradea, Romania; 11 Second Department of Pulmonology, Clinical Hospital of Infectious Diseases and Pulmonology “Victor Babes” Timisoara, Gheorghe Adam Street No. 13, 300310 Timisoara, Romania

**Keywords:** pleural infection, empyema, pleural fluid culture, microbiology, antimicrobial resistance

## Abstract

*Background and Objectives*: Pleural infection is heterogeneous, and the clinical meaning of a positive pleural culture remains uncertain. We compared adults with positive and negative aerobic pleural-fluid cultures and examined associations with major in-hospital events. *Materials and Methods*: This retrospective, single-center, case-ascertained cohort included adults hospitalized with bacterial pleural infection in Western Romania during 2017–2025. The primary analytic cohort was restricted to patients whose first clinically relevant pleural specimen had an interpretable aerobic culture. Pleural fluid (PF)-positive and PF-negative status was defined exclusively by that result; unperformed tests and operative specimens were not used for classification. The composite outcome comprised in-hospital death, intensive-care unit (ICU) admission/transfer, or thoracic surgery/surgical transfer. Prespecified covariates were entered simultaneously in logistic regression. *Results*: Of 317 included patients, 276 had an interpretable aerobic pleural culture: 127 (46.0%) were PF-positive and 149 (54.0%) were PF-negative. PF-positive patients were more often institutionalized, had chronic kidney disease, nosocomial infection, frank empyema, and lower pleural-fluid pH and glucose. Streptococci predominated in community-acquired infection, whereas Gram-negative organisms and multidrug-resistant phenotypes were more frequent in healthcare-associated/nosocomial episodes. The composite occurred in 60/127 (47.2%) PF-positive and 51/149 (34.2%) PF-negative patients (OR 1.72, 95% CI 1.06–2.80). The adjusted PF-positive estimate remained imprecise (aOR 1.60, 95% CI 0.94–2.74; *p* = 0.084), whereas healthcare-associated/nosocomial acquisition was associated with the composite (aOR 1.76, 95% CI 1.03–3.00; *p* = 0.038). In a sensitivity analysis restricted to 249 patients whose pleural sampling preceded all recorded outcome components, the adjusted association remained imprecise (aOR 1.54, 95% CI 0.86–2.76). *Conclusions*: A positive aerobic pleural culture identified a distinct high-risk phenotype and influenced antimicrobial management, but its independent association with the heterogeneous in-hospital composite remained uncertain. Acquisition setting and local resistance ecology should guide empirical therapy, and outcome timing should be reported explicitly in future studies.

## 1. Introduction

Pleural infection is a bacterial infection of the pleural space that most often develops as a complication of pneumonia, although non-pneumonic and healthcare-associated sources also occur. It represents a clinical continuum extending from complicated parapneumonic effusion, in which infected or highly inflammatory fluid requires drainage, to frank empyema, defined by macroscopically purulent pleural fluid. Because cultures are frequently negative, diagnosis cannot rely on microbiology alone and is supported by pleural-fluid acidity or hypoglycaemia, loculation or septation, and compatible clinical and radiological findings [1,2,3]. Progressive inflammation promotes fibrin deposition, compartmentalisation, and, in advanced disease, pleural organization that can restrict lung expansion.

Pleural infection remains an important cause of hospital admission, prolonged treatment, and death. Population-based studies have reported increasing incidence over recent decades, particularly among older men; in Finland, the overall incidence was 23.4 episodes per 100,000 person-years and rose during the study period [4]. Across published adult cohorts, approximately three-quarters of patients have at least one comorbidity, the median hospital stay is about 19 days, and reported mortality varies substantially according to population, case definition, and follow-up [5]. Large tertiary-care cohorts and prospective studies further show that adverse outcomes are concentrated among older patients and those with greater comorbidity, renal dysfunction, hypoalbuminaemia, or high Renal, Age, Purulence, Infection source, Dietary factors (RAPID) scores [6,7].

The clinical course is heterogeneous. Some patients improve with antibiotics and drainage, whereas others require repeated procedures, intrapleural therapy, intensive care, or thoracic surgery. Current management is based on prompt antimicrobial therapy and adequate source control, with image-guided chest-tube drainage for established complicated effusion or empyema [1,2]. In selected patients with poorly draining loculated infection, combined intrapleural tissue plasminogen activator and deoxyribonuclease improves radiographic clearance and can reduce surgical referral and length of stay [8]. Surgery remains necessary when medical source control fails or when advanced pleural organization prevents lung re-expansion. The relative use and timing of these strategies vary between centers, reflecting differences in case mix, expertise, and access to thoracic surgery [1,2].

Microbiological diagnosis is clinically important because pathogen identification may permit targeted antimicrobial therapy, yet conventional pleural-fluid culture has limited sensitivity. Previous antibiotic exposure, specimen handling, culture technique, anaerobic recovery, and the use of blood-culture bottles or molecular methods can materially influence diagnostic yield [9,10,11,12]. The causative spectrum also varies by geography and acquisition setting. Community-acquired disease is commonly associated with streptococci, oral anaerobes, and polymicrobial infection, whereas healthcare-associated or nosocomial disease more often includes *Staphylococcus aureus*, Enterobacterales, non-fermenting Gram-negative bacilli, and antimicrobial-resistant organisms [9,10,11]. Recent cohorts from North Africa and Southeast Asia have confirmed substantial regional differences in culture positivity, pathogen distribution, and resistance patterns, reinforcing the need for locally derived microbiological data [12,13]. A negative pleural culture therefore does not exclude pleural infection and may identify a clinically distinct, rather than simply less severe, subgroup.

Despite expanding evidence, several uncertainties remain. Studies comparing microbiologically positive and negative pleural infection have produced inconsistent findings. A recent bicentric cohort reported higher mortality among patients with pleural-fluid microbiological positivity [14], whereas another phenotyping study found important clinical differences but no independent association between culture positivity and mortality after adjustment [15]. A Danish multicenter cohort likewise observed similar short-term mortality in patients with known and microbiologically negative etiology [16], while a large surgical cohort found longer intensive-care and postoperative stays in culture-positive disease but similar short- and long-term survival [17]. These differences may reflect variation in referral pathways, sampling practices, acquisition setting, pathogen prevalence, treatment, and outcome definitions. Moreover, systematic reviews show that much of the available evidence comes from selected geographic regions and that microbiology is strongly context-dependent [5,9]. Contemporary, clinically integrated data from Romania and Eastern Europe remain limited, and international initiatives continue to identify regional variation in etiology, management, and outcomes as a major knowledge gap [18]. A well-characterized real-world cohort from this region can therefore provide information that is not readily transferable from other healthcare systems.

The primary objective was to compare the clinical, pleural-fluid, radiological, treatment, and in-hospital outcome profiles of adults with a positive versus negative aerobic culture from the first clinically relevant pleural specimen. Secondary objectives were to describe pathogen and resistance distributions by acquisition setting, quantify the timing and overlap of composite-outcome components, and estimate the adjusted association between PF-positive status and a major adverse in-hospital outcome.

## 2. Materials and Methods

### 2.1. Study Design and Setting

This was a single-center, retrospective observational cohort study with consecutive inclusion, conducted at the “Victor Babes” Infectious Diseases and Pneumoftiziology Hospital, a tertiary referral hospital that covers the population of four counties in western Romania. All adult admissions between 1 January 2017 and 31 December 2025 were screened. The unit of analysis was the index hospitalization for pleural infection; repeat admissions for the same episode were not counted separately. Follow-up extended from admission until discharge, transfer, or in-hospital death. The study was designed and reported in accordance with the STROBE statement and the RECORD extension for studies using routinely collected health data.

Candidate records were identified through a multisource search of discharge-diagnosis labels (empyema, pyothorax, pleural infection, complicated parapneumonic effusion, and parapneumonic effusion), pleural-fluid laboratory accessions, thoracentesis and chest-drain registers, ward admission/discharge lists, and thoracic-surgery transfer documentation. Candidates were linked by hospital identifier and admission dates and manually validated. The historical screening log retained the combined search concepts but not the exact diagnosis-code list, SQL syntax, or finer exclusion categories. Consequently, the cohort is described as case-ascertained rather than independently verifiable consecutive inclusion. Of 368 candidates, 51 were excluded under the single archived reason ‘source record unavailable or insufficient to verify eligibility or outcome’.

### 2.2. Study Population

Eligible patients were aged 18 years or older, had imaging-confirmed pleural effusion, and had a documented lower respiratory or pleural infectious syndrome treated with antibacterial therapy. For adjudication, a compatible syndrome required clinician-documented infection plus systemic inflammatory evidence (fever/hypothermia, leukocyte abnormality, elevated inflammatory markers, or sepsis) and respiratory/pleuritic symptoms or compatible pulmonary imaging. At least one pleural-infection criterion was also required: frank pus; positive pleural Gram stain or culture; pleural-fluid pH ≤ 7.20; pleural-fluid glucose ≤ 40 mg/dL (≤2.2 mmol/L); loculation/septation; pleural contrast enhancement, split-pleura sign, or intrapleural air in the appropriate clinical setting; or an explicit diagnosis of empyema/complicated parapneumonic effusion confirmed by clinical adjudication.

Exclusions were uncomplicated parapneumonic effusion, isolated tuberculous pleuritis without bacterial superinfection, non-infected malignant or other non-infectious effusion, isolated post-pneumonectomy empyema, repeat admission for a continuous unresolved episode, and insufficient information. A new episode required documented clinical/radiological resolution followed by a new infection; otherwise, only the first hospitalization was retained. Primary parapneumonic infection accompanied pneumonia and lacked a preceding pleural/thoracic procedure; postprocedural infection followed direct pleural instrumentation or thoracic surgery. Community-acquired infection had an event date no later than hospital day 2 without qualifying recent healthcare exposure. Healthcare-associated infection had community-onset timing plus at least one of: acute-care hospitalization for ≥2 days in the preceding 90 days; long-term-care residence; or intravenous therapy, chemotherapy, wound care, or hemodialysis in the preceding 30 days. Nosocomial infection had an event date on or after hospital day 3, with admission as day 1.

### 2.3. Data Sources and Collection

A standardized data dictionary was used to abstract electronic and paper records. Baseline clinical and serum laboratory values were the first available within 24 h of admission; pleural variables came from the first clinically relevant pleural sampling episode; treatments and outcomes covered the index hospitalization. A primary abstractor entered the data. Thirty-seven of 317 records (11.7%) underwent source-record re-verification during routine quality control; this check was not blinded, the selection mechanism was not retained in the audit log, and paired reviewer-level fields were unavailable, so inter-rater agreement could not be calculated. Thirty-three records flagged as requiring clinical adjudication were reviewed using the prespecified criteria; unresolved disagreements between two clinicians were referred to a third investigator. Range, date-order, logical-consistency, and cross-field concordance checks were applied before analysis.

#### 2.3.1. Clinical, Laboratory, and Imaging Variables

Collected variables included demographics, residence and institutionalization, comorbidities, Charlson Comorbidity Index, admission physiology, oxygen support, altered mental status, sepsis/septic shock, RAPID score, and routine serum measurements. Imaging was abstracted from the first relevant radiology report, with source-image review when needed. General findings included effusion size and laterality, loculation/septation, intrapleural air, consolidation, cavitation, malignancy suspicion, and fistula. Pleural effusion size was classified according to the original radiology report. On chest radiography, effusions explicitly described as large or massive were assigned to the corresponding category; no fixed quantitative threshold based on hemithorax occupation was applied. Size classification from other imaging modalities was likewise based on the corresponding radiology report. CT-specific findings were analyzed only when CT was recorded as the primary imaging modality; non-CT-primary records were treated as not evaluable rather than negative for pleural enhancement, split-pleura sign, and necrosis/abscess.

#### 2.3.2. Pleural-Fluid and Microbiological Assessment

Pleural specimens were obtained during routine care and submitted in a sterile container, inoculated into blood-culture bottles, or both (108, 75, and 93 patients, respectively, in the primary cohort). The recorded sampling-to-laboratory interval was available for 146/276 patients and was generally under 2 h. Gram stain and aerobic and anaerobic cultures were performed as ordered under the laboratory standard operating procedures in force at the time. Species identification and antimicrobial susceptibility results were abstracted from final validated reports. Susceptibility interpretation used the EUCAST clinical breakpoint table valid on the test date: versions 7.1 (2017), 8.0/8.1 (2018), 9.0 (2019), 10.0 (2020), 11.0 (2021), 12.0 (2022), 13.1 (2023), 14.0 (2024), and 15.0 (2025). Specific culture media, incubation durations, bottle brands, and identification/AST instrument models were not consistently recoverable across the nine-year period and were not inferred. For an exploratory comparison of aerobic culture positivity by specimen collection method, patients were grouped according to whether blood-culture bottles were used (alone or in combination with sterile-container submission) or specimens were submitted exclusively in sterile containers.

The qualifying specimen was the earliest pleural-fluid specimen during the index hospitalization with an interpretable aerobic culture. PF-positive status required a positive aerobic pleural culture, and PF-negative status required a negative result; an unperformed test was never coded as negative. Gram stain, anaerobic culture, blood/sputum/bronchoalveolar-lavage cultures, and operative specimens were descriptive and did not determine the primary exposure. Urinary antigen testing was not systematically captured in the study database and was therefore not included in the microbiological analyses. Seven operative specimens were positive, but all seven patients were already PF-positive from an earlier aerobic pleural culture; operative-only positivity was therefore zero. Polymicrobial infection required at least two organisms from the qualifying episode. MDR, XDR, and PDR followed the ECDC/CDC expert definitions: non-susceptibility to at least one agent in at least three categories; non-susceptibility in all but two or fewer categories; and non-susceptibility to all agents in all categories, respectively. ESBL and carbapenemase phenotypes were accepted only when explicitly reported after contemporaneous EUCAST-compliant testing; mechanisms were not inferred from susceptibility patterns. Common skin flora were considered pathogens only with a compatible sterile-site syndrome and concordant Gram stain, repeat isolation, or clinician adjudication; otherwise, they were excluded as probable contaminants. No PF-positive record was flagged as a probable contaminant. Pleural-fluid pH was abstracted from the final laboratory record. Syringe type, analyser model, documentation of air/local-anesthetic contamination checks, and the pH-specific processing interval were not retained consistently; no values were imputed, and this pre-analytic uncertainty was considered in interpretation.

#### 2.3.3. Treatment, Source Control, and Outcomes

Antimicrobial variables included the initial regimen, anaerobic and MRSA coverage, changes after microbiological results, concordance with the antibiogram, and intravenous/total treatment duration. Antibiotic exposure before pleural sampling was defined by documented pre-admission treatment or an in-hospital antibiotic start date earlier than the culture date; same-day order was not inferred. Source-control variables included thoracentesis, drain placement, type, caliber and guidance, repeat procedures, intrapleural therapy, VATS, thoracotomy, decortication, and transfer for thoracic surgery. Surgical transfer meant transfer to a thoracic-surgery service or hospital specifically for operative pleural source control. Operative procedure type after transfer was abstracted from available transfer/return documentation, but surgery dates were not retained.

The primary clinical outcome was a major adverse in-hospital composite comprising at least one of in-hospital death, ICU admission/transfer, or thoracic surgery/surgical transfer. ICU present on the admission date counted (*n* = 10). All events recorded during the index hospitalization counted in the principal association analysis; therefore, that analysis is phenotypic rather than strictly prognostic. Dates of the qualifying culture, ICU admission, surgical transfer, and death were compared at the patient level. Same-calendar-day sequence was classified as indeterminate. A strict-temporality sensitivity cohort excluded any patient whose qualifying sample was collected on or after a recorded component. A second sensitivity outcome counted only components occurring strictly after the sample. For transferred patients, follow-up for nonfatal events ended at transfer; post-transfer surgery type was captured when documented, whereas post-transfer ICU events and death were not systematically available. Component-specific results and overlap were prespecified as prominent secondary analyses.

### 2.4. Ethical Considerations

The study was conducted in accordance with the Declaration of Helsinki. The protocol was reviewed and approved by the Ethics Committee of the “Victor Babes” Hospital of Infectious Diseases and Pneumophysiology, Timisoara, Romania (approval no. 7595, 29 July 2026). Data were pseudonymized before analysis, the re-identification key was stored separately, and access was restricted to authorized investigators.

### 2.5. Statistical Analysis

No a priori sample-size calculation was performed because all verified eligible episodes were included. The 317-patient cohort was used for flow and case-definition summaries. The primary comparison was restricted to the 276 patients with an interpretable first aerobic pleural culture: 127 PF-positive and 149 PF-negative. Species and resistance analyses were restricted to PF-positive episodes with test-specific denominators. As an exploratory assessment of sample-size sensitivity, we estimated the minimum absolute difference in composite-event proportions detectable with 80% power using the fixed PF-positive and PF-negative group sizes, a two-sided significance level of 0.05, and the observed PF-negative event rate as the reference probability. This calculation used a normal approximation for two independent proportions without continuity correction and was not intended to assess power for the adjusted model.

Categorical variables are reported as n/N (%) and continuous variables as mean (standard deviation) or median [interquartile range]. Age, temperature, and systolic blood pressure were compared using Welch’s *t* test; other continuous variables used the Mann–Whitney U test. Categorical comparisons used Pearson’s chi-square or Fisher’s exact test when expected counts were sparse. Multicategory pathogen comparisons used the Fisher–Freeman–Halton test with Monte Carlo estimation (1,000,000 replicates; fixed seed 20260720). Unadjusted odds ratios (ORs) and 95% Wald confidence intervals (CIs) were calculated for clinically relevant binary comparisons.

The prespecified maximum-likelihood logistic model included age (per 10 years), sex, Charlson Comorbidity Index, healthcare-associated/nosocomial versus community acquisition, urea (per 10 mg/dL), albumin (per 0.5 g/dL decrease), C-reactive protein (per 50 mg/L), loculation/septation, purulent fluid, and PF-positive status. Covariates were entered simultaneously without automated selection. All 276 patients had complete model data; no imputation was used. Model convergence, separation, and functional form of continuous terms were assessed. Collinearity among model predictors was further evaluated using variance inflation factors (VIFs) calculated from the predictor matrix. Particular attention was given to healthcare-associated/nosocomial acquisition and serum albumin, with VIF values greater than 5 considered indicative of potentially problematic collinearity.

Sensitivity analyses restricted the PF-negative group to episodes without extrapleural microbiological evidence, restricted the cohort to primary parapneumonic infection, added admission year, required the qualifying sample to precede every recorded composite component, redefined the outcome to include only strictly post-sampling components, and expanded sampling eligibility to any interpretable aerobic or anaerobic pleural culture. The post-sampling and expanded-culture analyses were added in response to review and are exploratory. Component-specific and acquisition/phenotype analyses were unadjusted.

Missingness and evaluable denominators were reported for each variable. No outcome was imputed. All tests were two-sided, and *p* < 0.05 was considered statistically significant; secondary and sensitivity analyses were exploratory and no multiplicity correction was applied. Analyses were performed using SPSS version 26 (IBM Corp., Armonk, NY, USA).

## 3. Results

### 3.1. Cohort Assembly and Baseline Characteristics

Of 368 potentially eligible records, 51 were excluded because the source record was unavailable or insufficient to verify eligibility or outcome. The descriptive cohort comprised 317 patients. Forty-one had no interpretable aerobic pleural culture and were excluded from the primary comparison. The primary analytic cohort therefore included 276 patients: 127 (46.0%) PF-positive and 149 (54.0%) PF-negative. Among the 149 PF-negative patients, 19 had microbiological evidence from extrapleural specimens, whereas 130 had no recorded microbiological identification from the evaluated specimens. Extrapleural results did not alter the primary pleural-culture classification.

The cohort was predominantly male, with substantial comorbidity. Most infections were community-acquired, and loculation or septation was common. CT was the primary imaging modality in 139/276 (50.4%) patients. Detailed baseline characteristics are presented in Table 1, and data availability is summarized in Supplementary Table S1.

PF-positive patients were more frequently institutionalized (28/127 [22.0%] vs. 9/149 [6.0%]; *p* < 0.001), had higher Charlson scores (3 [2–5] vs. 2 [1–3]; *p* < 0.001), and more often had chronic kidney disease (38/127 [29.9%] vs. 20/149 [13.4%]; *p* < 0.001). Acquisition distribution differed (*p* = 0.008), principally because nosocomial infection occurred in 29/127 (22.8%) PF-positive versus 14/149 (9.4%) PF-negative patients. Frank empyema was also more frequent (75/127 [59.1%] vs. 62/149 [41.6%]; *p* = 0.004).

Among 215 patients with pleural biochemistry, PF-positive cases had lower pH (6.94 [6.78–7.11] vs. 7.20 [6.98–7.33]; *p* < 0.001) and glucose (23.5 [15.2–32.0] vs. 40.0 [28.0–51.0] mg/dL; *p* < 0.001). Consolidation was less frequent in PF-positive patients (81/127 [63.8%] vs. 115/149 [77.2%]; *p* = 0.014). CT-specific denominators did not show group differences in pleural enhancement, split-pleura sign, or necrosis/abscess (Table 1).

### 3.2. Microbiological Findings

Aerobic pleural culture was positive in 54/98 (55.1%) healthcare-associated/nosocomial and 73/178 (41.0%) community-acquired episodes (OR 1.77, 95% CI 1.08–2.89; *p* = 0.025). Within the primary cohort, Gram stain was positive in 120/230 (52.2%) tests performed and anaerobic culture in 38/213 (17.8%). Aerobic pleural-culture positivity was observed in 80/168 (47.6%) patients whose specimens were inoculated into blood-culture bottles, either alone or together with sterile-container submission, compared with 47/108 (43.5%) patients whose specimens were submitted exclusively in sterile containers (OR 1.18, 95% CI 0.73–1.92; *p* = 0.505). The difference was not statistically significant. This exploratory comparison should be interpreted cautiously because collection methods were determined by routine clinical practice rather than a standardized paired protocol (Table 2).

Antibiotic exposure before pleural sampling was present in 202/276 (73.2%) patients and did not differ by PF status or acquisition group. Blood-culture bottles were used for 168/276 (60.9%) pleural samples. Positive blood and sputum/bronchoalveolar-lavage cultures were documented in 70/276 (25.4%) and 53/276 (19.2%) patients, respectively.

Among 127 PF-positive episodes, 58 (45.7%) were Gram-positive, 54 (42.5%) were Gram-negative, and 15 (11.8%) were mixed. Streptococci accounted for 31 (24.4%), Enterobacterales for 30 (23.6%), other Gram-negative organisms for 19 (15.0%), Staphylococcus aureus for 18 (14.2%), mixed pathogens for 15 (11.8%), other organisms for 9 (7.1%), and anaerobes for 5 (3.9%). Enterobacterales or other Gram-negative organisms were recorded in 30/54 (55.6%) healthcare-associated/nosocomial versus 19/73 (26.0%) community-acquired PF-positive episodes.

Fifteen PF-positive episodes (11.8%) were polymicrobial and 107/127 (84.3%) had a full antibiogram. An MDR phenotype was recorded in 26/127 (20.5%), including 16/54 (29.6%) healthcare-associated/nosocomial and 10/73 (13.7%) community-acquired episodes (*p* = 0.028). MRSA occurred in 3/18 S. aureus episodes. Among 37 episodes with explicit marker results, ESBL and carbapenemase phenotypes were recorded in 10 (27.0%) and 7 (18.9%), respectively (Supplementary Table S2). Operational definitions used throughout the revised analyses—including cohort eligibility, PF classification, acquisition setting, microbiological and resistance phenotypes, surgical transfer, and timing-sensitive outcomes—are summarized in Supplementary Table S3.

### 3.3. Antimicrobial Treatment and Pleural Source Control

Grouped initial antimicrobial regimens did not differ significantly by PF status (*p* = 0.064). The most frequent individual regimens were ceftriaxone plus metronidazole (52/276 [18.8%]), piperacillin-tazobactam (51/276 [18.5%]), ampicillin-sulbactam (31/276 [11.2%]), and meropenem (31/276 [11.2%]). All patients received initial anaerobic coverage, and 59/276 (21.4%) received MRSA coverage. Regimens were changed in 76/127 (59.8%) PF-positive and 70/149 (47.0%) PF-negative patients (*p* = 0.033). Among 107 PF-positive episodes with an evaluable antibiogram, 79 (73.8%) regimens were concordant.

Median intravenous antibiotic duration was 14 [8–18] days and was longer in PF-positive than PF-negative patients (16 [12–21] vs. 11 [7–16] days; *p* < 0.001). Total duration was 24 [19–32] days overall, 28 [21–33] days in PF-positive patients, and 22 [18–30] days in PF-negative patients (*p* < 0.001).

Thoracentesis was performed in all 276 patients, and a chest drain was inserted in 242 (87.7%), with no difference by PF status. Among drained patients, the median time from admission to drainage was 1 [0–3] day, 53/242 (21.9%) required repositioning, and 58/242 (24.0%) underwent repeated drainage.

Overall intrapleural treatment use was similar between groups, but the choice of agent differed: combined tPA–DNase was more frequently used in PF-positive patients, whereas tPA alone was more common in PF-negative patients (Table 3).

VATS was recorded in 13/276 (4.7%), thoracotomy in 11/276 (4.0%), and decortication in 15/276 (5.4%). VATS and thoracotomy were mutually exclusive, and their union exactly matched the 24 surgical transfers; every transferred patient had one recorded operative approach, and decortication was nested within these procedures. Transfer frequency did not differ by PF status (12/127 [9.4%] vs. 12/149 [8.1%]; *p* = 0.682).

### 3.4. Clinical Outcomes and Length of Stay

The composite outcome occurred in 111/276 (40.2%) patients: 54 (19.6%) died in the hospital, 59 (21.4%) had ICU admission/transfer, and 24 (8.7%) underwent thoracic surgery/surgical transfer. Components overlapped; the first recorded component was ICU admission/transfer in 58 patients, surgical transfer in 19, and death in 34 (Supplementary Table S4).

The composite occurred in 60/127 (47.2%) PF-positive and 51/149 (34.2%) PF-negative patients (OR 1.72, 95% CI 1.06–2.80; *p* = 0.028). PF-positive patients had higher in-hospital death (34/127 [26.8%] vs. 20/149 [13.4%]; OR 2.36, 95% CI 1.28–4.35; *p* = 0.005) and ICU admission/transfer (36/127 [28.3%] vs. 23/149 [15.4%]; OR 2.17, 95% CI 1.20–3.90; *p* = 0.009). Surgical transfer did not differ (OR 1.19, 95% CI 0.52–2.75; *p* = 0.682).

The qualifying sample preceded ICU admission in 34/59 ICU events, occurred on the same day in 14, and followed ICU admission in 11; 10 ICU events were present on the admission date. The sample preceded surgical transfer in 23/24 events and occurred on the same day in one. It preceded death in 53/54 events and occurred on the same day in one. The strictly post-sampling composite occurred in 95/276 (34.4%) patients, including 54/127 (42.5%) PF-positive and 41/149 (27.5%) PF-negative patients (*p* = 0.009).

Median length of stay was 17 [12–28] days overall and did not differ by PF status. Mechanical ventilation was recorded in 68/276 (24.6%; *p* = 0.060 by PF status) and vasopressor support in 71/276 (25.7%; *p* = 0.043). Thirty-day mortality remained an exploratory available-case outcome because status was missing for a substantial proportion (Supplementary Table S1).

### 3.5. Factors Associated with the Major Adverse In-Hospital Outcome

**Figure 1:**
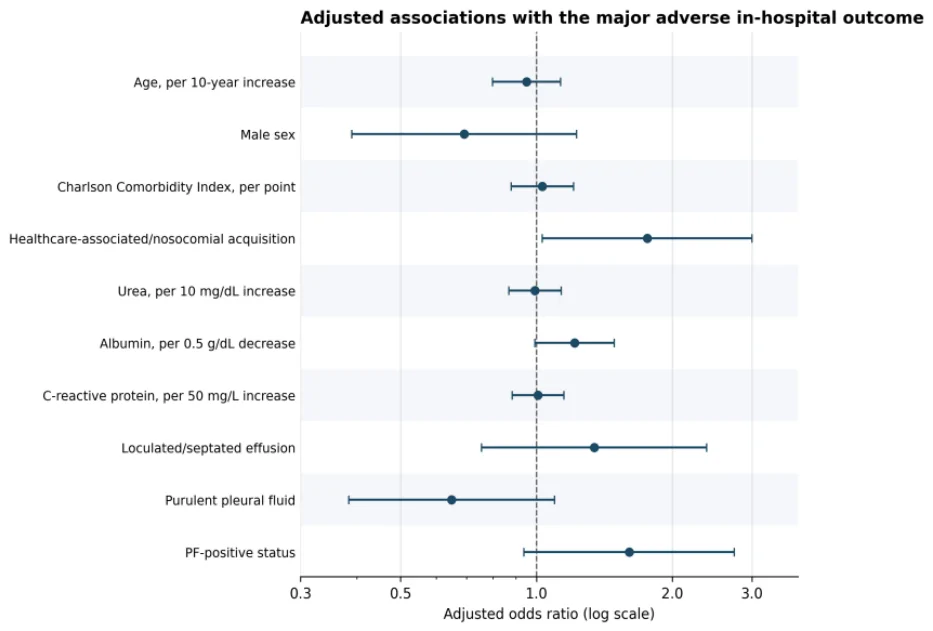
Adjusted associations with the major adverse in-hospital outcome in the primary analytic cohort.

The principal model included 276 patients and 111 events (Table 4; Figure 1). PF-positive status was associated with the composite before adjustment (OR 1.72, 95% CI 1.06–2.80; *p* = 0.028), while the adjusted estimate was 1.60 (95% CI 0.94–2.74; *p* = 0.084). The principal model describes associations across the index hospitalization and is phenotypic rather than strictly prognostic. Healthcare-associated/nosocomial acquisition was associated with the composite after adjustment (aOR 1.76, 95% CI 1.03–3.00; *p* = 0.038). The adjusted estimate per 0.5 g/dL albumin decrease was 1.21 (95% CI 0.99–1.49; *p* = 0.060). The overall likelihood-ratio test was *p* = 0.040. In an exploratory two-proportion sensitivity calculation, the available group sizes provided approximately 80% power to detect a 16.6-percentage-point difference in composite-event rates, compared with the observed difference of 13.0 percentage points. Collinearity diagnostics showed VIFs of 1.07 for healthcare-associated/nosocomial acquisition and 1.09 for serum albumin. The maximum VIF among all model predictors was 1.31, indicating no problematic multicollinearity.

PF-positive estimates remained directionally consistent after restriction to the strictly microbiology-negative reference (aOR 1.74, 95% CI 0.99–3.05), primary parapneumonic episodes (aOR 1.54, 95% CI 0.87–2.74), and adjustment for admission year (aOR 1.62, 95% CI 0.95–2.78). In the strict-temporality sensitivity analysis, which excluded patients whose qualifying pleural sample was collected on or after any recorded outcome component, 249 patients remained (84 events), yielding an aOR of 1.54 (95% CI 0.86–2.76; *p* = 0.145). When only strictly post-sampling components were counted, the exploratory aOR was 1.77 (95% CI 1.01–3.09; *p* = 0.045).

In secondary analyses, the composite occurred in 48/98 (49.0%) healthcare-associated/nosocomial and 63/178 (35.4%) community-acquired episodes (OR 1.75, 95% CI 1.06–2.89; *p* = 0.028), and in 50/137 (36.5%) frank empyema versus 61/139 (43.9%) non-purulent CPPE episodes (OR 0.73, 95% CI 0.45–1.19; *p* = 0.211). Within PF-positive episodes, the outcome did not differ materially between Gram-negative and Gram-positive infection or between polymicrobial and monomicrobial infection.

## 4. Discussion

### 4.1. Principal Findings and Clinical Interpretation

In this nine-year retrospective cohort, aerobic pleural-culture positivity identified a clinically distinct subgroup characterized by greater comorbidity, more frequent nosocomial acquisition and frank empyema, and more pronounced pleural biochemical abnormalities. PF-positive patients experienced higher unadjusted rates of adverse in-hospital outcomes, although the adjusted association between culture positivity and the composite outcome remained uncertain. Healthcare-associated/nosocomial acquisition retained an adjusted association with the composite outcome. Culture positivity was also accompanied by more frequent antibiotic modification and longer treatment durations, without clear differences in pleural drainage or surgical transfer. Overall, these findings suggest that aerobic culture positivity reflects a combination of host vulnerability, acquisition setting, and disease phenotype rather than functioning as an isolated prognostic determinant [14,15].

The phenotype associated with PF positivity extended beyond the microbiological result itself. Its association with greater comorbidity, institutionalization, nosocomial acquisition, purulence, and more marked pleural acidosis could reflect a combination of host vulnerability, greater bacterial burden, more advanced pleural inflammation, differences in organism cultivability, and the timing of presentation and sampling. Our data cannot distinguish between these mechanisms. In unadjusted analyses, the composite outcome occurred in 47.2% of PF-positive and 34.2% of PF-negative patients; in-hospital mortality was approximately twice as frequent in the PF-positive group, and intensive-care admission and vasopressor use were also more common. Nevertheless, the adjusted association with the composite outcome was smaller and statistically imprecise (aOR 1.60, 95% CI 0.94–2.74). Its direction was retained when the reference group was restricted to strictly microbiology-negative episodes (aOR 1.74, 95% CI 0.99–3.05) and when the analysis was restricted to patients whose qualifying culture preceded every recorded outcome component (aOR 1.54, 95% CI 0.86–2.76). In the exploratory analysis that counted only components occurring strictly after pleural sampling, the estimate was somewhat larger (aOR 1.77, 95% CI 1.01–3.09). These estimates should be interpreted in the context of their different temporal eligibility criteria and outcome definitions. The principal model included all 276 patients and 111 composite events recorded during the index hospitalization, including events that preceded or coincided with pleural sampling. Specifically, ICU admission preceded sampling in 11 patients and occurred on the same calendar day in 14, for whom the within-day sequence remained indeterminate. The strict-temporality sensitivity analysis restricted the cohort to 249 patients whose qualifying sample preceded every recorded outcome component (84 events), whereas the post-sampling analysis retained all 276 patients but counted only outcome components occurring strictly after sampling (95 events). These differences in patient eligibility and event counts may explain variations in the adjusted estimates and their precision. Accordingly, the modest differences between models should not be interpreted as evidence of a definitive change in the underlying association between PF-positive status and adverse outcomes.

This pattern lies between the findings of previous cohorts. Wong et al. similarly found that microbiologically positive patients had greater comorbidity and institutional residence but reported an independent association with mortality and substantially higher three-month mortality [14]. By contrast, Chan et al. observed higher unadjusted mortality among culture-positive empyema cases, but culture positivity was not independently associated with mortality after adjustment [15]. Meyer et al. found no material differences in mortality, intensive-care admission, surgery, or treatment duration between patients with microbiology-negative infection and those with a known bacterial etiology, although their definition of known etiology was broader than direct pleural-fluid positivity [16]. In a highly selected surgical population, Cheng et al. reported longer postoperative hospitalization, intensive-care stay, and ventilation among culture-positive patients, but no difference in 30-day mortality or two-year survival [17]. Differences in case selection, microbiological definitions, temporal ordering, outcome periods, and adjustment strategies limit direct comparison. Overall, the available evidence indicates that PF positivity defines a clinically meaningful risk phenotype but does not yet establish it as a sufficiently robust standalone prognostic determinant [14,15,16,17].

### 4.2. PF-Negative Pleural Infection and Diagnostiv Yield

PF-negative infection accounted for 149/276 (54.0%) episodes in the primary analytic cohort. Of these, 130 were strictly microbiology-negative, and 19 had compatible microbiological evidence only from extrapleural specimens. In the overall cohort, 73.2% had received antibiotics before pleural sampling, and blood-culture bottles were used for 60.9% of pleural samples. Neither exposure differed materially by PF status. These observations should not be interpreted as evidence that antibiotic treatment or specimen-processing methods do not influence microbiological yield. Antibiotic exposure was observationally measured, and treatment intensity, duration, and exact sequencing relative to same-day sampling could not be fully reconstructed; moreover, blood-culture bottles were used according to routine practice rather than in a protocolized paired comparison of the same specimen. In a prospective paired study, Menzies et al. showed that adding blood-culture bottle inoculation to standard pleural-fluid culture increased pathogen identification from 37.7% to 58.5% [19]. A recent population-based cohort also demonstrated an inverse association between the duration of previous antibiotic therapy and culture positivity, suggesting that the extent of exposure may be more informative than a binary exposure measure [20].

Molecular methods further demonstrate that conventional culture-negative disease often remains microbiologically identifiable. In 90 culture-negative specimens, 16S rRNA PCR detected a plausible pathogen in 31%, particularly fastidious and anaerobic organisms, and most causative positive results directly influenced management [21]. In a prospective study of clinically confirmed pleural infection, targeted metagenomic sequencing was positive in 70% of patients, compared with 35% for conventional culture, and identified additional species and polymicrobial anaerobic infections [22]. Similarly, the TORPIDS analysis found polymicrobial bacterial profiles in most sequenced pleural infections, considerably expanding the organism spectrum detected by culture [23]. PF-negative disease should therefore not be equated with microbiological sterility or necessarily with less advanced infection. It probably comprises a mixture of lower-burden disease, antibiotic-suppressed infection, fastidious or anaerobic infection, and episodes in which the available methods failed to identify the causative organism. The 19 episodes with extrapleural evidence also show why direct pleural-culture status and broader etiological identification should remain analytically distinct [19,20,21,22,23].

### 4.3. Microbial Ecology and Antimicrobial Resistance

The microbiological profile differed substantially by acquisition setting. Streptococci were the most frequent individual pathogen group, whereas Enterobacterales and other Gram-negative organisms were more common in healthcare-associated/nosocomial infections (55.6% vs. 26.0% of PF-positive episodes), alongside a higher prevalence of multidrug-resistant phenotypes (29.6% vs. 13.7%). These findings are consistent with previous studies demonstrating geographical and acquisition-related variation in pleural infection microbiology [9,10,12,13]. Conventional cultures probably underestimate anaerobic and polymicrobial infection, as demonstrated by molecular studies [11,23,24,25]. Importantly, the recorded ESBL and carbapenemase proportions refer only to the 37 episodes with explicit marker results and cannot be generalized to the entire cohort. These observations support acquisition-specific empirical antimicrobial therapy informed by local resistance patterns.

### 4.4. Antimicrobial Treatment and Source Control

Initial antimicrobial treatment was predominantly broad-spectrum and did not differ significantly between PF-positive and PF-negative patients, consistent with treatment being selected before pleural microbiological results became available. All patients received anaerobic coverage, while approximately one-fifth received empirical anti-MRSA therapy. By contrast, subsequent antibiotic modification was more frequent in PF-positive episodes, and treatment was concordant with the available antibiogram in 73.8% of evaluable cases. Microbiological identification therefore had a measurable influence on antimicrobial management, although maintaining broader coverage may have remained appropriate when polymicrobial infection, incompletely detected anaerobes, or concurrent extrapleural infection was suspected. Median intravenous and total treatment durations were 14 and 24 days, respectively, with PF-positive patients receiving longer courses than PF-negative patients. This difference probably reflects greater purulence, documented microbiology, comorbidity, or perceived disease severity rather than evidence that longer treatment is intrinsically more effective. In the SLIM trial, extending treatment from 14 to 21 to 28–42 days did not significantly reduce treatment failure among medically stabilized patients, while ODAPE suggested that two weeks of amoxicillin–clavulanate may be sufficient for carefully selected patients with stabilized community-acquired complicated parapneumonic effusion [26,27]. Both trials were small and enrolled selected populations, limiting their applicability to older, comorbid patients and those with nosocomial, resistant, or surgically managed infection.

Pleural drainage was performed in 87.7% of our cohort, generally within one day, and its use and timing were similar between microbiological groups. This supports source-control decisions based primarily on clinical, biochemical, and imaging evidence rather than culture status, as recommended by current guidance [1,2]. Available evidence also suggests broadly comparable outcomes with small-bore and large-bore drains, although dedicated randomized evidence remains limited [28]. Intrapleural treatment was administered to 46.7% of patients. Overall use was similar by PF status, although combined tPA-DNase was more frequent among treated PF-positive patients, and tPA alone was more frequent among treated PF-negative patients. MIST2 supports combined tPA-DNase, but not either agent alone [8]. Subsequent feasibility trials and meta-analyses have not yet established whether early intrapleural enzyme therapy or early VATS is generally superior: non-randomized studies suggest shorter hospitalization with VATS, but they are substantially affected by selection bias, while randomized studies have been too small for definitive efficacy comparisons [29,30,31]. Surgical transfer also did not differ by PF status. The exact equivalence between the 24 surgical transfers and the union of VATS and thoracotomy indicates complete overlap rather than separate additive events, with decortication nested within those procedures. Thus, treatment intensity in this cohort appears to have been driven mainly by the overall disease phenotype and response to initial drainage, whereas PF positivity had a clearer influence on antimicrobial modification and duration. Associations between individual interventions and outcomes cannot be interpreted causally because of confounding by indication.

### 4.5. Clinical Outcomes and Risk Stratification

The clinical burden of pleural infection was substantial, with 40.2% of patients experiencing the composite outcome. PF-positive patients had higher unadjusted event rates, but the adjusted association remained imprecise, with a confidence interval including unity. Sensitivity analyses yielded directionally consistent estimates of varying precision. These findings do not establish equivalence between PF-positive and PF-negative disease and reinforce the need for larger studies with standardized sampling and outcome definitions [5].

Healthcare-associated or nosocomial acquisition was the only covariate with a conventionally significant adjusted association with the composite outcome (aOR 1.76, 95% CI 1.03–3.00). This variable may integrate resistant or difficult-to-treat pathogens, recent healthcare exposure, frailty, delayed recognition, and referral complexity. Lower serum albumin retained a directionally adverse estimate, with an aOR of 1.21 per 0.5 g/dL decrement, but the 95% confidence interval included unity. This direction is concordant with the RAPID model, in which albumin, age, renal function, infection source, and pleural-fluid purulence jointly stratify mortality risk [32]. RAPID was derived in MIST1 and initially validated in MIST2, while the prospective PILOT cohort reported three-month mortality of 2.3%, 9.2%, and 29.3% across low-, intermediate-, and high-risk groups [7,32].

A subsequent Danish validation confirmed the gradient but reported corresponding risks of 2.4%, 17.0%, and 39.4%, illustrating that absolute risk varies between settings [33]. Albumin should therefore be regarded as an integrated marker of systemic inflammation, chronic illness, nutritional status, and physiological reserve, rather than as evidence that albumin replacement would alter outcome. Frank empyema was not associated with a higher composite-event rate in our secondary analysis, despite being more frequent among PF-positive patients. This is not necessarily paradoxical: purulence may prompt earlier recognition and drainage, and non-purulence, rather than purulence, contributes to the RAPID score [32].

Moreover, a multicenter CT cohort found no increase in 90-day mortality associated with loculation, whereas bronchopleural fistula carried a markedly less favorable prognosis [34]. Similarly, prospective PILOT analyses showed that sonographic septations influenced treatment decisions but were not independently related to mortality, surgery, or length of stay; pleural PAI-1, rather than septation itself, was associated with adverse outcomes [35]. Overall, host reserve, acquisition setting, and systemic illness may carry greater prognostic weight than purulence, loculation, or PF positivity considered in isolation. Neither albumin nor PF status should be used as a standalone decision rule, and the directionally consistent PF-positive estimates require evaluation in larger cohorts with standardized temporal assessment.

### 4.6. Clinical Implications

Empirical antimicrobial treatment should reflect the acquisition setting and local resistance patterns. Streptococci and oral flora remain important in community-acquired infection, whereas Gram-negative and resistant organisms are more frequent in healthcare-associated/nosocomial disease. Anaerobic coverage remains appropriate because conventional culture incompletely identifies anaerobic and polymicrobial infections [1,2,9,10,11,12,13,23,24,25].

An interpretable pleural specimen should be obtained as early as clinically feasible, ideally before additional antibiotic exposure or escalation of care, and its timing relative to subsequent outcomes should be documented. PF positivity may identify patients with greater host vulnerability, more advanced pleural biochemical disturbance, and increased unadjusted risk, justifying close clinical surveillance and microbiology-guided treatment refinement. Conversely, PF-negative status should not be equated with the absence of bacterial infection or used to justify delayed drainage, inappropriately narrow empirical therapy, or less intensive monitoring.

Albumin may contribute to initial risk assessment when interpreted alongside age, renal function, acquisition source, physiology, and the broader clinical phenotype, but neither our model nor the RAPID literature supports using albumin as an isolated decision rule [7,32,33]. Finally, death, intensive-care admission, and surgery should be reported separately even when a composite is used, because surgery may represent definitive source control rather than clinical deterioration, and access to intensive care or thoracic services is system-dependent.

The principal strengths of this study include a nine-year cohort, classification based on the first interpretable aerobic pleural culture, explicit separation of direct pleural positivity from extrapleural microbiological evidence, and integration of clinical, biochemical, imaging, microbiological, therapeutic, timing, and outcome data. Test-specific and variable-specific denominators, explicit operational definitions, component-level outcome accounting, and sensitivity analyses addressing microbiological reference groups and specimen-to-outcome timing further strengthen interpretability. The study also provides uncommon contemporary data from Romania and Eastern Europe, where detailed pleural-infection cohorts remain limited. Its contribution is therefore not to define a universal treatment algorithm, but to provide local epidemiological and resistance data that may inform empirical prescribing, microbiological sampling pathways, organization of pleural source control, and the design of future outcome studies. The need for comparable regional data is recognized in systematic reviews, the ERS/ESTS statement, and the international INTERMITTENT initiative [2,9,18].

### 4.7. Limitations and Future Research Directions

This study has limitations. Its retrospective, single-center design made case ascertainment and variable measurement dependent on existing clinical documentation. Exact diagnosis codes, query syntax, and granular reasons for the 51 exclusions were not preserved, so consecutive capture cannot be independently audited, and selection bias cannot be excluded. Generalizability to centers with different referral pathways, microbiological facilities, resistance patterns, or access to thoracic surgery is consequently limited. The primary analytic cohort also excluded 41 patients without an interpretable aerobic pleural culture. This restriction was necessary to define the exposure, but it creates a selected comparison population, and the findings should not be automatically extended to all patients with pleural infection.

Laboratory media, incubation duration, blood-culture-bottle brands, instrument platforms, and pH-specific pre-analytical procedures could not be reconstructed consistently across the nine-year period, although antimicrobial-susceptibility results were interpreted using the contemporaneous EUCAST standards applied by the hospital laboratory. Pre-sampling antibiotic exposure was common, blood-culture bottles were not used according to a uniform paired protocol, and anaerobic and molecular testing were incomplete. The PF-negative group is therefore etiologically heterogeneous, and misclassification resulting from antibiotic suppression, fastidious organisms, or limited diagnostic sensitivity may have attenuated between-group differences. CT-specific analyses used the conservative CT-primary denominator because the dataset did not identify every patient who underwent any CT.

The 37-record repeat check was not accompanied by a recoverable sampling scheme or reviewer-level agreement data, precluding a formal estimate of inter-rater reliability. Retrospective classification of community-acquired, healthcare-associated, and nosocomial episodes may also be imperfect despite the application of explicit operational criteria.

Clinical and laboratory practice, referral pathways, antimicrobial prescribing, use of tPA-DNase, and access to surgery or intensive care may have changed between 2017 and 2025. Adjustment for admission year did not materially alter the PF-positive estimate, but residual temporal confounding cannot be excluded. Treatment comparisons are particularly vulnerable to confounding by indication: patients receiving prolonged antibiotics, intrapleural therapy, or surgery are likely to differ systematically in disease severity, physiological reserve, microbiology, and response to initial drainage. The composite outcome also combines events with different clinical meanings. Death and intensive-care admission generally indicate adverse evolution, whereas surgery may represent both failure of initial medical treatment and successful definitive source control; intensive-care and surgical transfer are additionally influenced by eligibility, clinician judgment, bed availability, and local organization.

Although operative cultures did not create PF status and all seven operative-positive records were already positive on an earlier aerobic pleural culture, the principal exposure-outcome analysis was not fully prospective in temporal structure. Intensive-care admission preceded the qualifying sample in 11 patients, same-day sequence was indeterminate for some events, surgery dates were unavailable beyond transfer timing, and nonfatal follow-up ended at transfer. The post-sampling and strict-temporality analyses reduce particular sources of temporal ambiguity but use different event definitions or smaller effective samples and should be considered exploratory. Post-transfer intensive-care admission and death were not systematically captured. Thirty-day mortality was available only for a subset of patients, limiting the reliability of comparisons beyond hospitalization. Longer-term survival, including three-month mortality, could not be comprehensively assessed from the available records. Future prospective studies should incorporate standardized 30- and 90-day survival follow-up.

Finally, secondary and sensitivity analyses were exploratory without correction for multiple comparisons, several confidence intervals were wide, and the multivariable model was explanatory rather than a clinical prediction instrument and has not been externally validated.

In this Western Romanian cohort, PF positivity identified a distinct clinical, biochemical, and microbiological phenotype and was associated with greater unadjusted in-hospital morbidity and mortality. Its independent association with the composite outcome remained uncertain, whereas healthcare-associated or nosocomial acquisition was independently associated with adverse in-hospital outcomes and coincided with a greater burden of Gram-negative and multidrug-resistant organisms. Acquisition setting and local resistance ecology should inform empirical antimicrobial therapy, negative culture should not delay source control when pleural infection is otherwise established, and prospective multicenter studies using standardized sampling, molecular diagnostics, treatment pathways, temporal definitions, and harmonized component-level outcomes are required to validate these findings [2,18].

## 5. Conclusions

A positive aerobic pleural culture identified a clinically distinct subgroup with more comorbidity, nosocomial acquisition, frank empyema, severe pleural biochemical abnormalities, and higher unadjusted in-hospital risk. Its adjusted association with the composite outcome remained uncertain and was sensitive to how temporal eligibility and outcome timing were handled. Healthcare-associated/nosocomial acquisition was independently associated with the composite and coincided with a greater burden of Gram-negative and multidrug-resistant organisms. These findings support systematic early pleural sampling, acquisition-specific empirical therapy, explicit component-level outcome reporting, and prospective multicenter validation using standardized laboratory and timing definitions.

## Figures and Tables

**Table 1:** Baseline clinical, laboratory, pleural-fluid, and radiological characteristics according to aerobic pleural culture status.

Characteristic	Overall (*n* = 276)	PF-Positive (*n* = 127)	PF-Negative (*n* = 149)	*p* Value
Demographic characteristics and comorbidities				
Age, years	65.3 (14.9)	64.9 (16.3)	65.6 (13.6)	0.695
Male sex	204 (73.9%)	91 (71.7%)	113 (75.8%)	0.430
Institutionalized	37 (13.4%)	28 (22.0%)	9 (6.0%)	<0.001
Charlson Comorbidity Index	3 [1–4]	3 [2–5]	2 [1–3]	<0.001
Diabetes mellitus	80 (29.0%)	39 (30.7%)	41 (27.5%)	0.560
Chronic obstructive pulmonary disease	71 (25.7%)	32 (25.2%)	39 (26.2%)	0.853
Heart failure	42 (15.2%)	18 (14.2%)	24 (16.1%)	0.656
Chronic kidney disease	58 (21.0%)	38 (29.9%)	20 (13.4%)	<0.001
Active cancer	48 (17.4%)	25 (19.7%)	23 (15.4%)	0.353
Immunosuppression	38 (13.8%)	21 (16.5%)	17 (11.4%)	0.218
Aspiration risk	46 (16.7%)	25 (19.7%)	21 (14.1%)	0.214
Malnutrition	68 (24.6%)	34 (26.8%)	34 (22.8%)	0.448
Antibiotic exposure before pleural sampling, derived	202 (73.2%)	93 (73.2%)	109 (73.2%)	0.989
Infection phenotype and initial severity				
Infection acquisition				0.008
Community-acquired	178 (64.5%)	73 (57.5%)	105 (70.5%)	
Healthcare-associated	55 (19.9%)	25 (19.7%)	30 (20.1%)	
Nosocomial	43 (15.6%)	29 (22.8%)	14 (9.4%)	
Frank empyema	137 (49.6%)	75 (59.1%)	62 (41.6%)	0.004
Temperature, degrees C	37.7 (0.9)	37.6 (0.9)	37.8 (0.9)	0.100
Systolic blood pressure, mm Hg	123 (22.4)	122.9 (21.7)	123 (23)	0.987
Respiratory rate, breaths/min	22 [18–25]	21 [18–25]	22 [18–25]	0.719
Peripheral oxygen saturation, %	91 [87–95]	91 [87–95]	91 [88–95]	0.473
Altered mental status	50 (18.1%)	28 (22.0%)	22 (14.8%)	0.117
RAPID score	2 [1–3]	2 [1–3]	2 [1–2]	0.016
Laboratory findings				
White blood cell count, x10^9/L	11.7 [8.3–16.9]	11 [8.1–16.7]	12 [8.7–17.1]	0.378
C-reactive protein, mg/L	122 [71–191.2]	127 [73.5–192.5]	121 [71–191]	0.647
Albumin, g/dL	3 [2.6–3.4]	3 [2.6–3.4]	3.1 [2.7–3.5]	0.332
Urea, mg/dL	33 [25–44]	35 [25–44.5]	32 [24–43]	0.565
Creatinine, mg/dL	1.17 [0.85–1.9]	1.33 [0.82–2.19]	1.16 [0.85–1.71]	0.206
Pleural-fluid findings				
Pleural-fluid pH	7.06 [6.83–7.22]	6.94 [6.78–7.11]	7.2 [6.98–7.33]	<0.001
Pleural-fluid glucose, mg/dL	30 [19–41]	23.5 [15.2–32]	40 [28–51]	<0.001
Pleural-fluid LDH, U/L	690 [433.5–1155]	680.5 [434.5–1028.2]	728 [413–1212]	0.561
Radiological findings				
Large or massive pleural effusion	137 (49.6%)	68 (53.5%)	69 (46.3%)	0.231
Loculated or septated effusion	195 (70.7%)	91 (71.7%)	104 (69.8%)	0.736
Pleural thickening or enhancement, CT-primary subset	64/139 (46.0%)	24/57 (42.1%)	40/82 (48.8%)	0.437
Split pleura sign, CT-primary subset	48/139 (34.5%)	16/57 (28.1%)	32/82 (39.0%)	0.182
Air or hydropneumothorax	29 (10.5%)	13 (10.2%)	16 (10.7%)	0.892
Pulmonary consolidation	196 (71.0%)	81 (63.8%)	115 (77.2%)	0.014
Pulmonary necrosis or abscess, CT-primary subset	19/139 (13.7%)	9/57 (15.8%)	10/82 (12.2%)	0.544
Cavitation	35 (12.7%)	20 (15.7%)	15 (10.1%)	0.157

Data are n/N (%), mean (standard deviation), or median [interquartile range]. CT-specific variables use the CT-primary subgroup (overall *n* = 139; PF-positive *n* = 57; PF-negative *n* = 82); non-CT-primary records were not coded as negative. Denominators for pleural pH/glucose and LDH were 215 and 199, respectively. PF, pleural fluid; CT, computed tomography; LDH, lactate dehydrogenase.

**Table 2:** Pleural sampling, microbiological yield, pathogen distribution, and antimicrobial resistance according to infection acquisition.

Characteristic	Overall (*n* = 276)	Community-Acquired (*n* = 178)	Healthcare-Associated/ Nosocomial (*n* = 98)	*p* Value
Sampling procedures and microbiological yield				
Antibiotic exposure before pleural sampling	202 (73.2%)	131 (73.6%)	71 (72.4%)	0.837
Pleural sample inoculated in blood-culture bottles	168 (60.9%)	110 (61.8%)	58 (59.2%)	0.670
Positive Gram stain, among tests performed	120/230 (52.2%)	69/144 (47.9%)	51/86 (59.3%)	0.094
Positive aerobic pleural culture	127 (46.0%)	73 (41.0%)	54 (55.1%)	0.025
Positive anaerobic culture, among tests performed	38/213 (17.8%)	22/136 (16.2%)	16/77 (20.8%)	0.399
Positive blood culture	70 (25.4%)	43 (24.2%)	27 (27.6%)	0.535
Positive sputum or BAL culture	53 (19.2%)	33 (18.5%)	20 (20.4%)	0.706
Microbiological characteristics among PF-positive episodes				
Gram classification				0.029
Gram-positive	58 (45.7%)	40 (54.8%)	18 (33.3%)	
Gram-negative	54 (42.5%)	24 (32.9%)	30 (55.6%)	
Mixed	15 (11.8%)	9 (12.3%)	6 (11.1%)	
Polymicrobial infection	15 (11.8%)	9 (12.3%)	6 (11.1%)	0.834
Dominant pathogen group				0.019
Streptococcus spp.	31 (24.4%)	23 (31.5%)	8 (14.8%)	
Enterobacterales	30 (23.6%)	12 (16.4%)	18 (33.3%)	
Other Gram-negative organisms	19 (15.0%)	7 (9.6%)	12 (22.2%)	
Staphylococcus aureus	18 (14.2%)	12 (16.4%)	6 (11.1%)	
Mixed pathogens	15 (11.8%)	9 (12.3%)	6 (11.1%)	
Other organisms	9 (7.1%)	5 (6.8%)	4 (7.4%)	
Anaerobes	5 (3.9%)	5 (6.8%)	0 (0.0%)	
Full antibiogram available	107 (84.3%)	65 (89.0%)	42 (77.8%)	0.085
Recorded MDR phenotype	26 (20.5%)	10 (13.7%)	16 (29.6%)	0.028
MRSA, among S. aureus episodes with explicit results	3/18 (16.7%)	2/12 (16.7%)	1/6 (16.7%)	1.000
ESBL, among episodes with explicit marker results	10/37 (27.0%)	3/14 (21.4%)	7/23 (30.4%)	0.710
Carbapenemase, among episodes with explicit marker results	7/37 (18.9%)	3/14 (21.4%)	4/23 (17.4%)	1.000

PF-positive denotes a positive aerobic culture from the first clinically relevant pleural specimen. Tests not performed are excluded from test-specific denominators. Pathogen and resistance rows are restricted to PF-positive episodes. MDR, multidrug-resistant; MRSA, methicillin-resistant Staphylococcus aureus; ESBL, extended-spectrum beta-lactamase; BAL, bronchoalveolar lavage.

**Table 3:** Antimicrobial treatment, pleural source control, timing-sensitive outcomes, and resource use according to aerobic pleural culture status.

Characteristic	Overall (*n* = 276)	PF-Positive (*n* = 127)	PF-Negative (*n* = 149)	*p* Value
Antimicrobial treatment				
Initial antibiotic regimen				0.064
Non-antipseudomonal beta-lactam with anaerobic coverage	103 (37.3%)	48 (37.8%)	55 (36.9%)	
Antipseudomonal beta-lactam regimen	73 (26.4%)	35 (27.6%)	38 (25.5%)	
Carbapenem monotherapy	48 (17.4%)	22 (17.3%)	26 (17.4%)	
Vancomycin-containing broad-spectrum regimen	33 (12.0%)	19 (15.0%)	14 (9.4%)	
Fluoroquinolone plus metronidazole	19 (6.9%)	3 (2.4%)	16 (10.7%)	
Initial anaerobic coverage	276 (100.0%)	127 (100.0%)	149 (100.0%)	--
Initial MRSA coverage	59 (21.4%)	27 (21.3%)	32 (21.5%)	0.965
Antibiotic regimen changed	146 (52.9%)	76 (59.8%)	70 (47.0%)	0.033
Concordant with antibiogram, among evaluable episodes	79/107 (73.8%)	79/107 (73.8%)	--	--
Intravenous antibiotic duration, days	14 [8–18]	16 [12–21]	11 [7–16]	<0.001
Total antibiotic duration, days	24 [19–32]	28 [21–33]	22 [18–30]	<0.001
Pleural drainage and intrapleural treatment				
Thoracentesis performed	276 (100.0%)	127 (100.0%)	149 (100.0%)	--
Chest drain inserted	242 (87.7%)	111 (87.4%)	131 (87.9%)	0.896
Time from admission to first drainage, days	1 [0–3]	1 [0–3]	1 [0–3]	0.917
Drain type, among drained patients				0.309
Small-bore (<14 Fr)	146 (60.3%)	71 (64.0%)	75 (57.3%)	
Pigtail	30 (12.4%)	10 (9.0%)	20 (15.3%)	
Large-bore (≥14 Fr)	66 (27.3%)	30 (27.0%)	36 (27.5%)	
Drain caliber, Fr	12 [12–20]	12 [12–20]	12 [12–20]	0.642
Drain guidance, among drained patients				0.760
Ultrasound	126 (52.1%)	55 (49.5%)	71 (54.2%)	
Computed tomography	55 (22.7%)	27 (24.3%)	28 (21.4%)	
Blind	61 (25.2%)	29 (26.1%)	32 (24.4%)	
Number of drains, among drained patients	2 [1–2]	1 [1–2]	2 [1–2]	0.329
Drain repositioning, among drained patients	53/242 (21.9%)	19/111 (17.1%)	34/131 (26.0%)	0.098
Repeated drainage, among drained patients	58/242 (24.0%)	27/111 (24.3%)	31/131 (23.7%)	0.905
Any intrapleural treatment	129 (46.7%)	60 (47.2%)	69 (46.3%)	0.877
Intrapleural agent, among treated patients				0.034
tPA plus DNase	77 (59.7%)	41 (68.3%)	36 (52.2%)	
tPA alone	26 (20.2%)	6 (10.0%)	20 (29.0%)	
DNase alone	11 (8.5%)	7 (11.7%)	4 (5.8%)	
Other	15 (11.6%)	6 (10.0%)	9 (13.0%)	
Number of intrapleural doses, among treated patients	3 [2–4]	3 [1–4]	3 [2–4]	0.172
Number of pleural procedures	3 [2–3]	3 [2–3]	3 [2–3]	0.660
Surgical source control				
Video-assisted thoracoscopic surgery	13 (4.7%)	9 (7.1%)	4 (2.7%)	0.085
Thoracotomy	11 (4.0%)	3 (2.4%)	8 (5.4%)	0.203
Decortication	15 (5.4%)	9 (7.1%)	6 (4.0%)	0.264
Surgical transfer	24 (8.7%)	12 (9.4%)	12 (8.1%)	0.682
Clinical outcomes and resource use				
Major adverse in-hospital outcome	111 (40.2%)	60 (47.2%)	51 (34.2%)	0.028
Post-sampling composite outcome, exploratory	95 (34.4%)	54 (42.5%)	41 (27.5%)	0.009
In-hospital death	54 (19.6%)	34 (26.8%)	20 (13.4%)	0.005
ICU admission or transfer	59 (21.4%)	36 (28.3%)	23 (15.4%)	0.009
Thoracic surgery or surgical transfer	24 (8.7%)	12 (9.4%)	12 (8.1%)	0.682
Mechanical ventilation	68 (24.6%)	38 (29.9%)	30 (20.1%)	0.060
Vasopressor support	71 (25.7%)	40 (31.5%)	31 (20.8%)	0.043
Length of stay, days	17 [12–28]	16 [11–29]	18 [13–27]	0.667
Length of stay among in-hospital survivors, days	20 [14–32.8]	21 [14–35]	20 [14–29]	0.307
ICU stay among ICU patients, days	8 [5–12]	8 [4–10.5]	10 [7.5–13.5]	0.106
Mechanical ventilation duration among ventilated patients, days	5 [3–7]	5 [3–8.5]	5 [2.2–6]	0.247
Vasopressor duration among treated patients, days	4 [2–5.5]	3 [1.8–5]	4 [3–6]	0.030

The post-sampling composite counts only components dated strictly after pleural culture; same-day events were excluded. All 24 surgical transfers overlapped exactly with either VATS (*n* = 13) or thoracotomy (*n* = 11); these approaches were mutually exclusive, and decortication (*n* = 15) was nested. PF, pleural fluid; ICU, intensive care unit; VATS, video-assisted thoracoscopic surgery; tPA, tissue plasminogen activator; DNase, deoxyribonuclease.

**Table 4:** Univariable and multivariable associations with the major adverse in-hospital outcome, with sensitivity analyses.

Predictor	Unadjusted OR	95% CI	*p* Value	Adjusted OR	95% CI	*p* Value
Age, per 10-year increase	0.98	0.83–1.15	0.824	0.95	0.80–1.13	0.571
Male sex	0.73	0.43–1.26	0.259	0.69	0.39–1.23	0.208
Charlson Comorbidity Index, per point	1.10	0.96–1.26	0.156	1.03	0.88–1.21	0.710
Healthcare-associated/nosocomial acquisition	1.75	1.06–2.89	0.028	1.76	1.03–3.00	0.038
Urea, per 10 mg/dL increase	0.98	0.87–1.12	0.812	0.99	0.87–1.13	0.915
Albumin, per 0.5 g/dL decrease	1.26	1.04–1.52	0.018	1.21	0.99–1.49	0.060
C-reactive protein, per 50 mg/L increase	1.01	0.89–1.15	0.826	1.01	0.88–1.15	0.909
Loculated/septated effusion	1.30	0.76–2.22	0.336	1.34	0.76–2.38	0.315
Purulent pleural fluid	0.73	0.45–1.19	0.211	0.65	0.38–1.10	0.106
PF-positive status	1.72	1.06–2.80	0.028	1.60	0.94–2.74	0.084
Sensitivity analyses for PF-positive status						
**Sensitivity analysis**	**N**	**Events**		**Adjusted OR**	**95% CI**	***p* value**
Primary analysis	276	111		1.60	0.94–2.74	0.084
Strict microbiology-negative reference	257	103		1.74	0.99–3.05	0.052
Primary parapneumonic episodes only	242	100		1.54	0.87–2.74	0.139
Additional adjustment for admission year	276	111		1.62	0.95–2.78	0.078
Qualifying sample before every recorded component	249	84		1.54	0.86–2.76	0.145
Post-sampling composite outcome	276	95		1.77	1.01–3.09	0.045
Any evaluable pleural culture (aerobic or anaerobic)	296	116		1.69	1.00–2.84	0.050

## Data Availability

The data presented in this study are available on request from the corresponding author. The data are not publicly available due to legal and ethical considerations.

## Supplementary Materials

The following supporting information can be downloaded at: https://www.mdpi.com/article/10.3390/medicina62102013/s1, Figure S1: Study flow and derivation of the analytic populations; Table S1: Data availability and evaluable denominators; Table S2: Detailed microbiological findings and recorded antimicrobial-resistance phenotypes; Table S3: Operational definitions used in the revised analysis; Table S4: Timing of the qualifying pleural culture and overlap among outcome components.

## Abbreviations

The following abbreviations are used in this manuscript:

AKI	Acute Kidney Injury
aOR	Adjusted Odds Ratio
CI	Confidence Interval
CKD	Chronic Kidney Disease
CRP	C-Reactive Protein
CT	Computed Tomography
DNAse	Deoxyribonuclease
ICU	Intensive Care Unit
IET	Intrapleural Enzyme Therapy
LOS	Length of Stay
MDR	Multidrug-Resistant
MRSA	Methicillin-Resistant *Staphylococcus aureus*
OR	Odds Ratio
PAI-1	Plasminogen Activator Inhibitor-1
PCR	Polymerase Chain Reaction
PF	Pleural Fluid
PF-negative	Pleural Fluid Microbiology-Negative
PF-positive	Pleural Fluid Microbiology-Positive
RAPID	Renal (urea), Age, Purulence, Infection source, Dietary factors (serum albumin)
tPA	Tissue Plasminogen Activator
VATS	Video-Assisted Thoracoscopic Surgery
