# Supplementary material for: Pleural Aerobic Culture Status, Clinical Phenotypes, and In-Hospital Outcomes in Adult Pleural Infection: A Retro-Spective Cohort Study
Source: Medicina (Kaunas). 2026 Oct 18;62(10):2013. doi: 10.3390/medicina62102013 (PMC13707034; doi:10.3390/medicina62102013)
Supplement: Supplementary file 1 [file medicina-62-02013-s001.zip › medicina-4536545-supplementary.pdf]

Pleural Aerobic Culture Status, Clinical Phenotypes, and In-Hospital Outcomes in Adult Pleural Infection: A Retrospective Cohort Study

Supplementary File

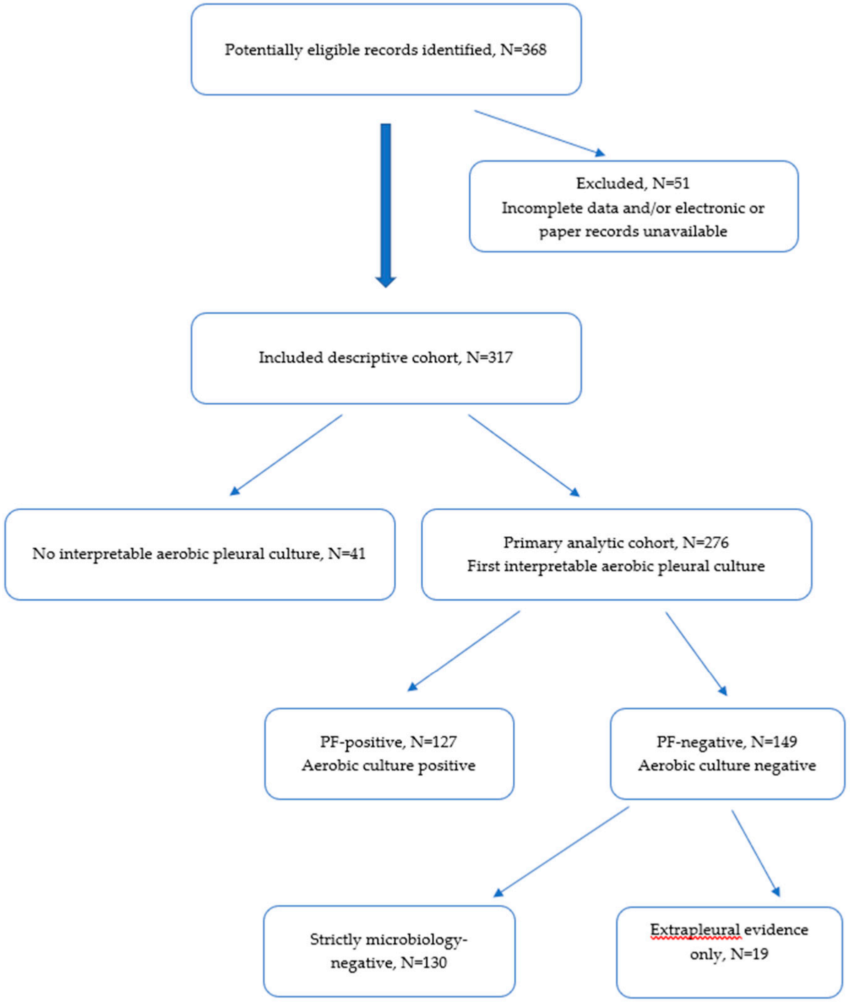

Supplementary Figure S1. Study flow and derivation of the analytic populations.
PF status was defined exclusively by the first interpretable aerobic pleural culture. Unperformed aerobic cultures and operative specimens were not used for the primary classification. PF, pleural fluid.

Supplementary Table S1. Data availability and evaluable denominators.

Variable	Analysis population	Available/evaluable n/N (%)	Missing/not performed n/N (%)
Age	Primary analytic cohort	276/276 (100.0%)	0/276 (0.0%)
Sex	Primary analytic cohort	276/276 (100.0%)	0/276 (0.0%)
Institutionalization	Primary analytic cohort	276/276 (100.0%)	0/276 (0.0%)
Charlson Comorbidity Index	Primary analytic cohort	276/276 (100.0%)	0/276 (0.0%)
Infection acquisition	Primary analytic cohort	276/276 (100.0%)	0/276 (0.0%)
Empyema versus CPPE	Primary analytic cohort	276/276 (100.0%)	0/276 (0.0%)
Derived antibiotic exposure before pleural sampling	Primary analytic cohort	276/276 (100.0%)	0/276 (0.0%)
Temperature	Primary analytic cohort	276/276 (100.0%)	0/276 (0.0%)
Systolic blood pressure	Primary analytic cohort	276/276 (100.0%)	0/276 (0.0%)
Respiratory rate	Primary analytic cohort	276/276 (100.0%)	0/276 (0.0%)
Peripheral oxygen saturation	Primary analytic cohort	276/276 (100.0%)	0/276 (0.0%)
Altered mental status	Primary analytic cohort	276/276 (100.0%)	0/276 (0.0%)
RAPID score	Primary analytic cohort	276/276 (100.0%)	0/276 (0.0%)
White blood cell count	Primary analytic cohort	276/276 (100.0%)	0/276 (0.0%)
C-reactive protein	Primary analytic cohort	276/276 (100.0%)	0/276 (0.0%)
Albumin	Primary analytic cohort	276/276 (100.0%)	0/276 (0.0%)
Urea	Primary analytic cohort	276/276 (100.0%)	0/276 (0.0%)
Creatinine	Primary analytic cohort	276/276 (100.0%)	0/276 (0.0%)
Pleural-fluid pH	Primary analytic cohort	215/276 (77.9%)	61/276 (22.1%)
Pleural-fluid glucose	Primary analytic cohort	215/276 (77.9%)	61/276 (22.1%)
Pleural-fluid LDH	Primary analytic cohort	199/276 (72.1%)	77/276 (27.9%)
Purulent pleural fluid	Primary analytic cohort	276/276 (100.0%)	0/276 (0.0%)
Effusion volume	Primary analytic cohort	276/276 (100.0%)	0/276 (0.0%)
Loculation or septation	Primary analytic cohort	276/276 (100.0%)	0/276 (0.0%)
CT designated as primary imaging modality	Primary analytic cohort	139/276 (50.4%)	137/276 (49.6%)
Pleural thickening or enhancement	CT-primary subset	139/139 (100.0%)	0/139 (0.0%)
Split pleura sign	CT-primary subset	139/139 (100.0%)	0/139 (0.0%)
Necrosis or abscess	CT-primary subset	139/139 (100.0%)	0/139 (0.0%)

Variable	Analysis population	Available/evaluable n/N (%)	Missing/not performed n/N (%)
Air or hydropneumothorax	Primary analytic cohort	276/276 (100.0%)	0/276 (0.0%)
Pulmonary consolidation	Primary analytic cohort	276/276 (100.0%)	0/276 (0.0%)
Cavitation	Primary analytic cohort	276/276 (100.0%)	0/276 (0.0%)
Gram stain result (test performed)	Full included cohort	260/317 (82.0%)	57/317 (18.0%)
Aerobic culture result (test performed)	Full included cohort	276/317 (87.1%)	41/317 (12.9%)
Anaerobic culture result (test performed)	Full included cohort	233/317 (73.5%)	84/317 (26.5%)
Sampling-to-laboratory interval	Primary analytic cohort	146/276 (52.9%)	130/276 (47.1%)
Full antibiogram available	PF-positive episodes	107/127 (84.3%)	20/127 (15.7%)
Initial antibiotic regimen	Primary analytic cohort	276/276 (100.0%)	0/276 (0.0%)
Chest drain insertion	Primary analytic cohort	276/276 (100.0%)	0/276 (0.0%)
Surgical transfer date	Patients transferred for surgery	24/24 (100.0%)	0/24 (0.0%)
ICU admission date	Patients admitted/transferred to ICU	59/59 (100.0%)	0/59 (0.0%)
Death date	In-hospital deaths	54/54 (100.0%)	0/54 (0.0%)
Length of stay	Primary analytic cohort	276/276 (100.0%)	0/276 (0.0%)
Exploratory 30-day mortality status	Primary analytic cohort	201/276 (72.8%)	75/276 (27.2%)

Availability is reported within the population relevant to each analysis. CT-specific variables are restricted to the CT-primary subgroup, and non-CT-primary records are not coded as negative. Pleural tests coded as not performed are unavailable, not negative. CPPE, complicated parapneumonic pleural effusion; CT, computed tomography; ICU, intensive care unit; LDH, lactate dehydrogenase; PF, pleural fluid.

Supplementary Table S2. Detailed microbiological findings and recorded antimicrobial-resistance phenotypes.

Microbiological finding	Overall PF-positive (n=127)	Community-acquired (n=73)	Healthcare-associated/ nosocomial (n=54)
**Organism recorded as the principal pleural isolate or dominant pathogen**			
Streptococcus anginosus group	26/127 (20.5%)	18/73 (24.7%)	8/54 (14.8%)
Streptococcus pneumoniae	5/127 (3.9%)	5/73 (6.8%)	0/54 (0.0%)
Staphylococcus aureus	18/127 (14.2%)	12/73 (16.4%)	6/54 (11.1%)
Enterococcus faecium	2/127 (1.6%)	2/73 (2.7%)	0/54 (0.0%)
Enterococcus faecalis	1/127 (0.8%)	1/73 (1.4%)	0/54 (0.0%)
Klebsiella pneumoniae	20/127 (15.7%)	8/73 (11.0%)	12/54 (22.2%)
Escherichia coli	10/127 (7.9%)	4/73 (5.5%)	6/54 (11.1%)
Pseudomonas aeruginosa	12/127 (9.4%)	5/73 (6.8%)	7/54 (13.0%)
Acinetobacter baumannii	7/127 (5.5%)	2/73 (2.7%)	5/54 (9.3%)
Fusobacterium nucleatum	3/127 (2.4%)	3/73 (4.1%)	0/54 (0.0%)
Prevotella spp	2/127 (1.6%)	2/73 (2.7%)	0/54 (0.0%)
Mixed oral flora / polymicrobial	15/127 (11.8%)	9/73 (12.3%)	6/54 (11.1%)
Other	6/127 (4.7%)	2/73 (2.7%)	4/54 (7.4%)
**Polymicrobial infection and resistance phenotypes**			
Polymicrobial infection	15/127 (11.8%)	9/73 (12.3%)	6/54 (11.1%)
Second-organism species recorded, among polymicrobial episodes	8/15 (53.3%)	4/9 (44.4%)	4/6 (66.7%)
Full antibiogram available	107/127 (84.3%)	65/73 (89.0%)	42/54 (77.8%)
Recorded MDR phenotype	26/127 (20.5%)	10/73 (13.7%)	16/54 (29.6%)
MRSA, among S. aureus episodes with explicit results	3/18 (16.7%)	2/12 (16.7%)	1/6 (16.7%)
ESBL, among episodes with explicit marker results	10/37 (27.0%)	3/14 (21.4%)	7/23 (30.4%)
Carbapenemase, among episodes with explicit marker results	7/37 (18.9%)	3/14 (21.4%)	4/23 (17.4%)

Species-level counts use the recorded principal pleural isolate/dominant pathogen and sum to 127 PF-positive episodes. Polymicrobial episodes are counted once in the mixed category. Resistance markers use marker-specific evaluable denominators. ESBL, extended-spectrum beta-lactamase; MDR, multidrug-resistant; MRSA, methicillin-resistant Staphylococcus aureus; PF, pleural fluid.

Supplementary Table S3. Operational definitions used in the revised analysis.

Term	Operational definition	Data/source note
Index hospitalization	First hospitalization for a continuous unresolved pleural-infection episode; a new episode required documented resolution followed by a new infection.	Patient identifier and admission dates
Compatible infectious syndrome	Clinician-documented lower respiratory/pleural infection treated with antibiotics, with systemic inflammatory evidence plus respiratory/pleuritic symptoms or compatible pulmonary imaging.	Manual source-record adjudication
Compatible structural imaging	Loculation/septation, pleural contrast enhancement, split-pleura sign, or intrapleural air in a clinically infected patient.	CT-specific variables evaluated only in CT-primary records
First qualifying pleural specimen	Earliest pleural-fluid sampling episode during the index hospitalization with an interpretable aerobic culture.	Culture collection date
PF-positive	Positive aerobic culture from the first qualifying pleural specimen.	Gram stain, anaerobic, extrapleural, and operative results did not define exposure
PF-negative	Negative aerobic culture from the first qualifying pleural specimen.	Unperformed tests excluded
Strictly microbiology-negative	PF-negative with no recorded extrapleural microbiological evidence.	Sensitivity-analysis reference group
Representative operative specimen	Pleural tissue/fluid obtained during VATS/thoracotomy and recorded descriptively.	Not used for PF classification; operative-only n=0
Primary parapneumonic	Pleural infection accompanying pneumonia without preceding pleural/thoracic procedure or direct inoculation.	Sensitivity analysis
Postprocedural	Pleural infection following direct pleural instrumentation or thoracic surgery.	Classified separately
Community-acquired	Event no later than hospital day 2 and no qualifying recent healthcare exposure.	Admission date is hospital day 1
Healthcare-associated	Community-onset timing plus >=2-day hospitalization in prior 90 days, long-term-care residence, or IV therapy/chemotherapy/wound care/hemodialysis in prior 30 days.	Source-record exposure history
Nosocomial	Event date on or after hospital day 3.	Contemporaneous HAI timing rule
Frank empyema	Macroscopically purulent pleural fluid.	Gross appearance
CPPE	Eligible non-purulent pleural infection meeting biochemical, microbiological, imaging, or adjudicated criteria.	Complicated parapneumonic pleural effusion
Polymicrobial infection	At least two organisms recovered from the qualifying pleural episode.	Species-level second-organism data could be incomplete
MDR/XDR/PDR	Non-susceptibility to >=1 agent in >=3 categories / all but <=2 categories / all agents in all categories.	Magiorakos et al.; XDR/PDR not separately analyzable in the retained dataset
ESBL/carbapenemase	Accepted only when explicitly reported by the laboratory after contemporaneous EUCAST-compliant testing.	No inference from susceptibility pattern
Probable contaminant	Typical skin flora without concordant Gram stain, repeat isolation, or compatible clinical adjudication.	No PF-positive record met this definition

Term	Operational definition	Data/source note
Major adverse composite	At least one in-hospital death, ICU admission/transfer, or thoracic surgery/surgical transfer during the index hospitalization.	ICU present on admission counted
Surgical transfer	Transfer to a thoracic-surgery service/hospital specifically for operative pleural source control.	Procedure type captured when documented; surgery date unavailable
Strict-temporality cohort	Qualifying culture strictly before every recorded composite component.	Same-day sequence excluded
Post-sampling composite	Composite counting only components dated strictly after the qualifying culture.	Exploratory sensitivity outcome

Definitions were applied to the retained source-record fields. HAI, healthcare-associated infection; ICU, intensive care unit; MDR, multidrug-resistant; PF, pleural fluid; VATS, video-assisted thoracoscopic surgery.

Supplementary Table S4. Timing of the qualifying pleural culture and overlap among outcome components.

Event/overlap	n	Culture before event	Same calendar day	Culture after event
ICU admission/transfer	59	34	14	11
Surgical transfer	24	23	1	0
In-hospital death	54	53	1	0
**Surgery/transfer overlap**				
VATS	13			
Thoracotomy	11			
VATS and thoracotomy	0			
VATS or thoracotomy	24			
Decortication (nested within VATS/thoracotomy)	15			
Surgical transfer	24			
Surgery without documented transfer	0			
Transfer without documented VATS/thoracotomy	0			
**Composite-component overlap**				
Death only	33			
ICU only	36			
Surgical transfer only	18			

Event/overlap	n	Culture before event	Same calendar day	Culture after event
Death and ICU	18			
Death and surgical transfer	1			
ICU and surgical transfer	3			
All three components	2			
**First recorded composite component**				
ICU admission/transfer	58			
Surgical transfer	19			
In-hospital death	34			

For event-timing rows, the denominator is the number experiencing that event. Same-day sequence was not inferred. Surgery dates were unavailable; the surgical transfer date was used. The first recorded composite component had no same-day ties in the retained dates.
